# Cuckoo Search Algorithm Based on Repeat-Cycle Asymptotic Self-Learning and Self-Evolving Disturbance for Function Optimization

**DOI:** 10.1155/2015/374873

**Published:** 2015-08-03

**Authors:** Jie-sheng Wang, Shu-xia Li, Jiang-di Song

**Affiliations:** ^1^School of Electronic and Information Engineering, University of Science and Technology Liaoning, Anshan 114044, China; ^2^National Financial Security and System Equipment Engineering Research Center, University of Science and Technology Liaoning, Anshan 114044, China

## Abstract

In order to improve convergence velocity and optimization accuracy of the cuckoo search (CS) algorithm for solving the function optimization problems, a new improved cuckoo search algorithm based on the repeat-cycle asymptotic self-learning and self-evolving disturbance (RC-SSCS) is proposed. A disturbance operation is added into the algorithm by constructing a disturbance factor to make a more careful and thorough search near the bird's nests location. In order to select a reasonable repeat-cycled disturbance number, a further study on the choice of disturbance times is made. Finally, six typical test functions are adopted to carry out simulation experiments, meanwhile, compare algorithms of this paper with two typical swarm intelligence algorithms particle swarm optimization (PSO) algorithm and artificial bee colony (ABC) algorithm. The results show that the improved cuckoo search algorithm has better convergence velocity and optimization accuracy.

## 1. Introduction

Cuckoo search (CS) algorithm, a new biological heuristic algorithm, is put forward by Yang and Deb in 2009. It simulates the cuckoo's seeking nest and spawning behavior and introduces Levy flight mechanism into it, which is able to quickly and efficiently find the optimal solution [1, 2]. Studies have proved that CS algorithm is better than other swarm intelligence algorithms in convergence rate and optimization accuracy, such as genetic algorithm (GA), particle swarm optimization (PSO) algorithm, and artificial bee colony (ABC) algorithm [3]. Because this algorithm has the characteristics of fewer parameters and being simple and easy to implement, now it has been successfully applied in a variety of engineering optimization problems. So the CS algorithm has a very high potential research value [4, 5].

CS algorithm is a new type of bionic algorithm. Many scholars carry out many researches in CS algorithm and put forward the corresponding improvement strategies. In literature [6], it gains insight into search mechanisms of CS algorithm and analyzes why it is efficient; meanwhile, it discusses the essence of algorithms and its link to self-organizing systems [6]. In literature [7], in order to increase CS efficiency, it exploits several parameters of the CS algorithm involving the Levy distribution factor beta (*β*) and the probability factor (*P*) and by seeking optimum values of these parameters efficiency of CS algorithm are improved [7]. In literature [8], it studied the algorithmic structure and behavior of CS and Levy distribution in detail, and then by comparing with widely used optimization algorithms (i.e., DE and GA) statistical results verified that CS has a more superior problem-solving ability [8]. For the purpose of enhancing the search ability of the cuckoo search (CS) algorithm, an improved robust approach, called harmony search (HS), is put forward, in which method a mutation operator is added to the process of the cuckoos updating to speed up convergence [9]. In literature [10], in order to solve combinatorial problems, it extends and improves CS by reconstructing its population and introducing a new category of cuckoos [10]. A cuckoo optimal algorithm based on the exchange operator and the chaotic disturbance is proposed, which introduces the exchange operator theory of the particle swarm optimization algorithm to improve convergence rate and optimization accuracy [11]. A cooperative coevolutionary cuckoo search algorithm is put forward by applying the framework of cooperative coevolutionary, which divides the solution vectors of population into several subvectors and constructs the corresponding subswarms [12]. A novel cuckoo search optimization algorithm based on Gauss distribution is proposed by adding a Gauss distribution to CS algorithm to improve convergence rate [13]. A new self-adaptive cuckoo search algorithm is proposed by using a self-adaptive parameter control strategy to adjust the step size of CS to enhance its search ability [14]. Because the CS algorithm is highly random search according to Levy flight mechanism and shows a strong leaping, it is easy to jump from one region to another region, which makes the search that around each bird's nest location not careful and thorough and cannot make full use of information nearby the bird's nest locations. So the CS algorithm has characteristics of weak local searching ability, slow convergence rate, and low optimization accuracy.

In order to make up the defect of the algorithm on this aspect, a kind of improved cuckoo search algorithm based on the repeat-cycle asymptotic self-learning and self-evolving disturbance (RC-SSCS) is proposed. In order to obtain better disturbance effect, the learning and updating strategy of the worst frog in the shuffled frog leaping algorithm (SFLA) and part of differential evolution (DE) thought are introduced into the constructing of disturbance factor, thus, which makes every disturbance with the effect of nest's self-learning and self-evolving. Finally, the six typical test functions are chosen for simulation experiment and the simulation results proved that the improved cuckoo search algorithm has better convergence rate and optimization accuracy. The paper is organized as follows. In Section 2, the cuckoo search algorithm is introduced. The improved cuckoo search algorithm based on the repeat-cycle asymptotic self-learning and self-evolving disturbance is presented in Section 3. In Section 4, the simulation experiments and results analysis are introduced in details. Finally, the conclusion illustrates the last part.

## 2. Cuckoo Search Algorithm

Cuckoo search algorithm (CS) is developed by Xin-she Yang by observing the magical nature phenomenon and then giving an artificial processing, which is a new type of heuristic search algorithm [15, 16]. This algorithm is mainly based on two aspects: the cuckoo's parasitic reproduction mechanism and Levy flights search principle. In nature, cuckoos use a random manner or a quasirandom manner to seek bird's nest location [10]. Most of cuckoos lay their eggs in other bird nests and let the host raise their cubs instead of them. If a host found that the eggs are not its owns, it will either throw these alien eggs away from the nest or abandon its nest and build a new nest in other places. However, there are some cuckoos choosing nest that the color and shape of the host's egg are similar with their own to win the host's love, which can reduce the possibility of their eggs being abandoned and increase the reproduction rate of the cuckoos.

In general, each cuckoo can only lay one egg and each egg on behalf of one solution (cuckoo). The purpose is to make the new and potentially better solutions replace the not-so-good solutions (cuckoos). In order to study the cuckoo search algorithm better, the simplest method is adopted, that is to say, only one egg is in each nest. In this case, an egg, a bird's nest, or a cuckoo is no different, which is to say, each nest corresponding to a cuckoo's egg. For simplicity in describing the cuckoo search algorithm, Yang and Deb use the following three idealized rules to construct the cuckoo algorithm [17].

(1) Each cuckoo only lays one egg at a time and randomly chooses bird's nest to hatch the egg.
(2) The best nest will carry over to the next generation.
(3) The number of available host nests is fixed, and the probability of a host discovering an alien egg is *P*_*a*_ = [0,1]. In this case, the host bird may either throw the alien egg away or abandon its nest so as to build a new nest in a new location.

Under the above conditions, the specific steps of CS algorithm are described as follows.

*(1) Initialization Setting*. Randomly generate *N* bird's nest location *X*_0_ = (*x*_1_^0^, *x*_2_^0^,…, *x*_*N*_^0^), and then take the *N* bird's nest positions into test functions for choosing experiments. Through testing, the best bird's nest location is chosen and carried over to the next generation.

*(2) Searching of Bird's Nest Location*. Equation (1) is used to realize the location update and search the bird's nest location of the next generation in order to gain a new set of bird's nest locations, which are taken into the test function again for testing experiments. After comparing with the last generation nest locations, the best bird's nest location is chosen and entered into the next step. Consider

(1) $$\begin{array}{c} x_{i}^{\left(t+1\right)}=x_{i}^{t}+\alpha ⊕Levy\left(\lambda \right),\;i=1,2,\ldots ,n. \end{array}$$

*(3) Selection of Bird's Nest Locations*. The probability of a host discovering an alien egg *P*_*a*_ = 0.25 is compared with the random number that obeys uniform distribution *r* ∈ [0,1]. If *r*, the value of *x*_*i*_^*t*+1^ is randomly changed. Otherwise it does not need to be changed.

Then the changed bird's nest locations are calculated with the test functions, which are compared with the optimal position of previous generation and best bird's nest locations *X*_*t*_ = (*x*_1_^*t*^, *x*_2_^*t*^,…, *x*_*N*_^*t*^) is recorded. Finally, the optimal nest position *pb*_*t*_^*∗*^ is chosen.

*(4) Accuracy or Iterations Judgment*. Calculate and judge whether *f*(*pb*_*t*_^*∗*^) achieves the object accuracy or the terminating conditions. If it meets the requirements, *pb*_*t*_^*∗*^ is the global optimal solution *gb*; if it is not be met, *pb*_*t*_^*∗*^ is kept to the next generation and return to step (2) and start the next loop iteration and update again.

According to these four steps described above of cuckoo search algorithm, the cuckoo algorithm not only uses the Levy flight (global search) search method, but also introduces the elite reserved strategy (local search), which makes the algorithm have both global search ability and local search ability. The purpose of step (3) in this algorithm is to increase the diversity of solution so that the algorithm is prevented from being caught into local optimum and achieving the global optimum.

The search path of CS algorithm is different with other swarm algorithms. The CS algorithm uses Levy flight, which has character of strong randomness. Broadly speaking, Levy flight is a random walk, whose step size obeys Levy distribution, and the direction of travel is subject to uniform distribution. The step size vector of CS algorithm is determined by Mantegna rule of Levy distribution characteristics. In Mantegna rules, the step size *s* is designed as follows:

(2) $$\begin{array}{c} s=\frac{u}{{\left|v\right|}^{1/\beta }}, \end{array}$$

where *u* and *v* obey the normal distribution, that is to say

(3) $$\begin{array}{c} u\sim N\left(0,\sigma_{u}^{2}\right),\;\;v\sim N\left(0,\sigma_{v}^{2}\right), \end{array}$$

where

(4) $$\begin{array}{c} \sigma_{v}={\left\{\frac{\Gamma \left(1+\beta \right)\sin\left(\pi \beta /2\right)}{\Gamma \left[\left(1+\beta \right)/2\right]\beta 2^{\left(\beta -1\right)/2}}\right\}}^{1/\beta },\\ \sigma_{u}=1. \end{array}$$

But here the chosen method of the direction obeys uniform distribution. The searching pattern of CS algorithm is Levy flight, for instance, the *i*th cuckoo of the *t*th generation generates the next generation solution *x*_*i*_^*t*+1^:

(5) $$\begin{array}{c} x_{i}^{t+1}=x_{i}^{t}+\alpha ⊕Levy\left(\lambda \right), \end{array}$$

where ⊕ is a point to point multiplication and the step size of Levy(*λ*) obeys the Levy distribution, which can be expressed as follows:

(6) $$\begin{array}{c} Levy\sim u=t^{-\lambda },\;\left(1<\lambda \leq 3\right). \end{array}$$

Here, the Mantegna rules are used to calculate the step size. In (5), *α* is the step controlled quantity mainly used to control the direction and step size. In that its distribution is a power function, Levy distribution has infinite variance and its increment obeys heavy-tailed distribution [18, 19]. Levy flight is seemingly Brownian motion under the status of a long-distance flight, or it may be described that Levy flight consists of frequent short-jump and occasional long-jump. The long-jump can help the CS algorithm jump out of local optimum. Consider

(7) $$\begin{array}{c} \alpha =O\left(\frac{L}{10}\right), \end{array}$$

where *L* is the search space size of optimization problems.

Thus, the generation of some new solutions is gradually through the Levy flight and rand walk around the optimal solution to obtain the optimal solution, which can speed up the local searching. On the contrary, a part of new solutions is far away from the current optimal solution, because they are randomly generated by deviating from remote locations. The main purpose of these solutions is to ensure that the system does not fall into the local optimal solution.

A large number of simulation experiments prove that when the bird's nest groups values *n* = 15~40 and the detection probability *P*_*a*_ = 0.25, it can solve many optimization problems [16]. Once the population size *n* is fixed, the discovery probability *P*_*a*_ is an important parameter to balance the global search and the local search and control the elite selection. Therefore, the CS algorithm has characteristic of less parameters, excellent searching path and strong global optimization ability, and so forth [20].

## 3. The Improved Cuckoo Search Algorithm

For each time, the length and direction of cuckoos' searching path are highly randomly changed based on Levy flight mechanism, so they are easy to jump from one region to another, which is beneficial to the global search in the early stage of optimization and make the CS algorithm have strong global search ability [16, 21]. It is just because the CS algorithm shows a strong jumping in the search process that makes the local search around each bird's nest location no careful and no thorough. Therefore, the local optimization information near bird's nest location has not been fully utilized, which leads to that the local search ability is not strong, the optimization accuracy of the later period is not high, and the convergence speed is slow. In order to improve the convergence velocity and the optimization accuracy of the CS algorithm, a self-learning and self-evolving disturbance operation is added to the algorithm, and a further study for the improvement of disturbance is also discussed.

### 3.1. Cuckoo Search Algorithm Based on Self-Learning and Self-Evolving Disturbance

In order to make the algorithm carry on more careful and thorough searches near bird's nest locations, after each iteration of CS algorithm a set of obtained preponderant bird's nest locations *X*_*i*_(*t*) instead of letting *X*_*i*_(*t*) directly into the next iteration, a disturbance operation is applied to it for making a further search on the neighborhood of *X*_*i*_(*t*).

Due to the general disturbance, such as Gauss perturbation and random perturbation, all having the great randomness and blindness, in order to obtain a better disturbance effect, the learning and updating strategy of the worst frog in the shuffled frog leaping algorithm (SFLA) and a part of differential evolution (DE) thought are introduced into the constructing of the disturbance factor. The introduction of learning and updating strategy of the worst frog can increase bird's self-learning ability of each bird's nest learning from the optimal nest [22]. That is to say it can increase the speed of other solutions approaching to the best solution and improve the algorithm's convergence rate. The introduction of the differential evolution thought can increase the diversity of bird's nest location, which makes every bird's nest have the evolution ability. The good learning and evolving ability are bound to cause high search ability.

Based on the above analysis, the disturbance factor is constituted of two parts: one part is the learning factor from SFLA learning and updating strategy of the worst frog; the other part is the evolution factor based on a part of differential evolution (DE) thoughts. Thus, the whole disturbance factor makes every disturbance with the effect of bird's self-learning and self-evolving ability. Disturbance factor is structured as follows:

(8) $$\begin{array}{c} \varepsilon =c_{1}u_{1}\left(x_{i}-x_{best}\right)+c_{2}u_{2}\left(x_{r_{1}}-x_{r_{2}}\right), \end{array}$$

where *x*_*i*_ is the *i*th disturbed bird's nest location, *x*_best_ is the current best location, *r*_1_ and *r*_2_ are random number from (1, *n*), *r*_1_ ≠ *r*_2_, *n* is the number of bird's nest population, *x*_*r*_1__ and *x*_*r*_2__ are the bird's nest locations corresponded to a random number *r*_1_ and *r*_2_, *u*_1_, and *u*_2_ obey the Gaussian distribution, *c*_1_ is the learning scale, and *c*_2_ is evolution scale.

For better controlling of the disturbance range, a controlled quantity of disturbance scope *γ* is introduced to control the search scope size around a bird's nest. After being disturbed, the bird's nest location is express as

(9) $$\begin{array}{c} x_{i}^{\prime }=x_{i}+\gamma ⊕\varepsilon , \end{array}$$

where *x*_*i*_′ is the bird's nest location after being disturbed, *γ* is the controlled quantity of the disturbance range, and ⊕ is the point to point multiplication.

According to (8) and (9), after searching and selection operation obtain a bird's nest location *x*_*i*_, do not let this nest go into the next generation directly. Instead, add to it a disturbance that take *x*_*i*_ as the foundation and *ε* disturbance factor, within the distribute scope that is controlled by *γ*. Finally after disturbance get a new bird's nest location *x*_*i*_′, and let *x*_*i*_′ go into the next generation.

In different stages, the bird nest's learning ability, the evolving ability, and the disturbance scope can be controlled by adjusting the values of *c*_1_, *c*_2_, and *γ*. For example, when *γ* is a given value, if *c*_1_ = 0 and *c*_2_ is a certain constant, then within this scope of search, the bird's nest only has evolution ability, without learning ability; by the same token, if *c*_2_ = 0 and *c*_1_ is a certain constant, within the search scope, the algorithm only has learning ability and no evolving ability.

The disturbance range controller *γ* not only controls the search scope size, but also affects a bird nest's learning ability and evolution ability. When *γ* is given a big value, the coefficient before learning factor *x*_*i*_ − *x*_best_ and evolving factor *x*_*r*_1__ − *x*_*r*_2__ can be driven larger, so the learning and evolution ability become stronger. Under this condition, the search scope of the algorithm is larger, the search ability is stronger but the search fineness is lower. By the same token, when *γ* is given a small value, the learning and evolution ability are relatively weaker, the search scope is smaller, the search ability becomes weaker, but the search fineness is much higher.

### 3.2. Cuckoo Search Algorithm Based on Repeat-Cycle Asymptotic Self-Learning and Self-Evolving Disturbance

The ideal disturbance effect should be that in the early stage of disturbances, it has higher searching ability and in the later stage of disturbances has higher search accuracy. In this way, it can obtain a better bird's nest location fast and then carry on a more careful and thorough search around the better nest location. Based on the above analysis, a repeat-cycle asymptotic disturbance method is proposed. Adopting a dynamic adjustment *γ* makes the search scope gradually change from big to small after disturbance. In other words, based on the results of last disturbance, narrowing the disturbance scope, go on the next disturbance. The repeat-cycle asymptotic disturbance search is carried out in turn.

In order to obtain a better bird's nest position at a faster speed, at the beginning of disturbance *γ* is given a larger value. With the disturbance continuing, the bird's nests gradually get better and the adjustment of bird's nest locations is more and more subtle. Particularly in the condition that the response of fitness value is sensitive to parameter changing, it needs to use a very small amount of control *γ* to make the position have a fine-tuning near optimal value. The *γ* is adjusted according to the following:

(10) $$\begin{array}{c} \gamma =\gamma_{min}+\left(\gamma_{max}-\gamma_{min}\right)\times \frac{N-n}{N}, \end{array}$$

where *γ*_min_ is the minimum value of control amount of disturbance scope, *γ*_max_ is the maximum value, *N* is the total number of repeat-cycle disturbances, and *n* is the *n*th disturbance.

In the circulation disturbance, the controlled quantity of the disturbance range *γ* is controlled by disturbance number *n*. Make *γ* changes between *γ*_max_ and *γ*_min_. When *n* = 1, *γ* is maximum *γ* = *γ*_max_. With the disturbance number *n* increasing gradually *γ* decrease little by little. Finally when *n* = *N*, *γ* reaches minimum value *γ* = *γ*_min_.

In the early stage of the repeat-cycle disturbances, the bird's nest self-learning and self-evolving function plays a leading role, which can make it surely find a better bird's nest faster. With disturbance number increasing, the search scope gradually turns smaller and the fineness degree of search is gradually enhanced. In the later stage of the repeat-cycle disturbances, the fineness search plays a leading role.

### 3.3. Algorithm Procedure

**Figure 1:**
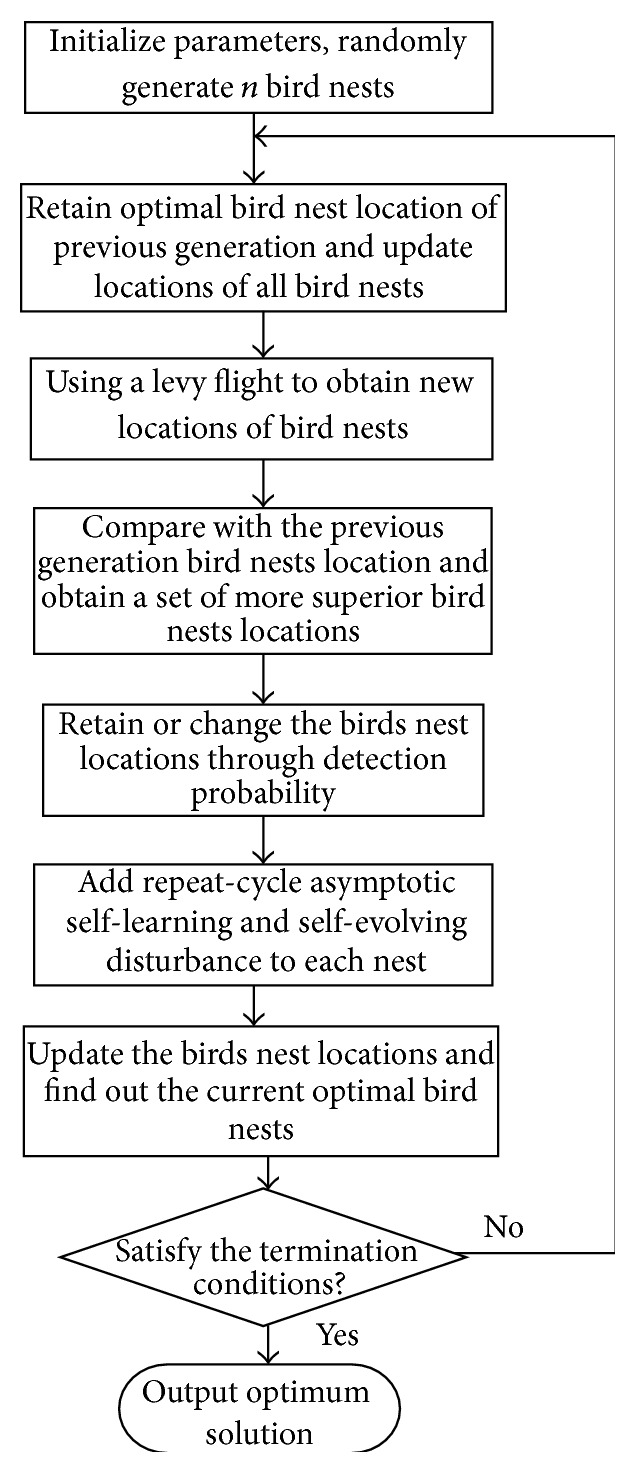
Flow chart of RC-SSCS algorithm.

The algorithm procedure of the improved cuckoo search algorithm (RC-SSCS) is shown in Figure 1. The specific steps of RC-SSCS algorithm are described as follows.

**Step 1 (initialization).**

Randomly generate *N* bird's nest location *X* ^0^ = (*x*_1_^0^, *x*_2_^0^,…, *x*_*N*_^0^), select the best bird's nest location, and carry it over to the next generation.

**Step 2 (searching operation).**

Use the location update (1) to search for the next generation bird's nest position, obtain a new set of bird's nests, and test them. Then compare the testing results with the bird's nest position of previous generation and obtain a set of better positions.

**Step 3 (selection operation).**

Generate the random number *r* ∈ [0,1], which obeys the uniform distribution. Contrast it with the detection probability *P*_*a*_ = 0.25, if *r* > *P*_*a*_, *x*_*i*_^(*t*+1)^ is changed randomly, otherwise *x*_*i*_^(*t*+1)^ is unchanged. Test the changed nest positions, compare them with locations of the last step, and choose the better nest locations.

**Step 4 (repeat-cycle disturbance operation).**

Add a self-learning and self-evolving disturbance to each bird's nest location, test new bird's nest locations that have been disturbed, and then compare the test results with locations of the last disturbance results and choose the better bird's nest locations. After many times disturbance, obtain a set of the best bird's nest locations, and then choose a best location *pb* from the set.

**Step 5 (judgment operation).**

Calculate the fitness value of *f*(*pb*) and judge whether it achieves the termination condition. If it is satisfied, *pb* is the optimal solution, otherwise return to Step 2 and start the next iteration.

## 4. Simulation Results and Analysis

**Figure 2:**
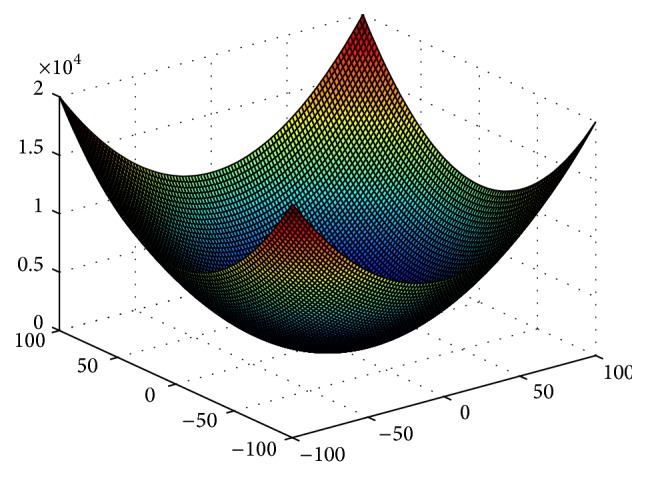
3D surface figure of Sphere function.

**Figure 3 fig3:**
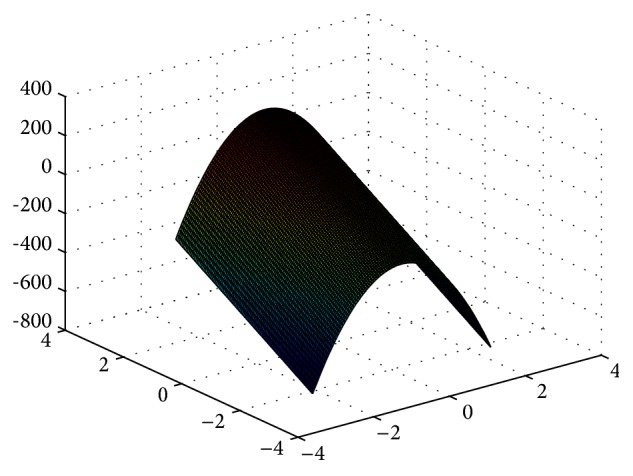
3D surface figure of Rosenbrock function.

**Figure 4 fig4:**
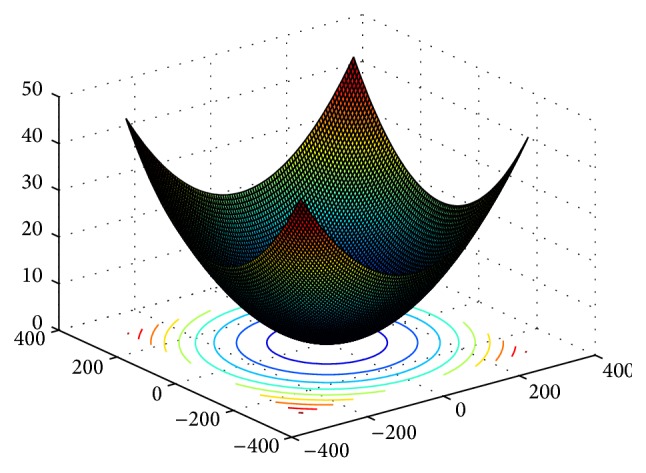
3D surface figure of Griewank function.

**Figure 5 fig5:**
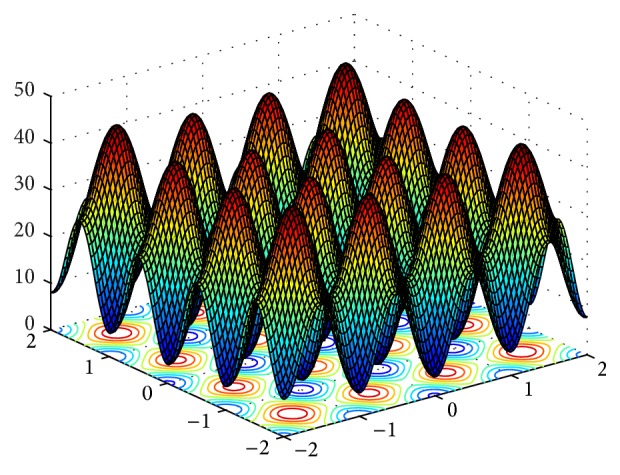
3D surface figure of Rastrigrin function.

**Figure 6 fig6:**
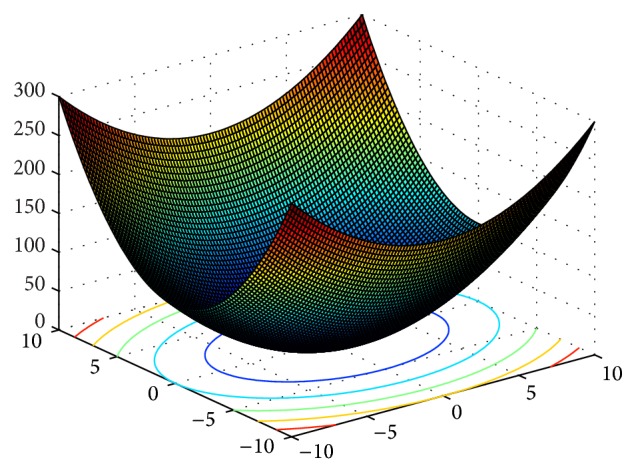
3D surface figure of Sumsquares function.

**Figure 7:**
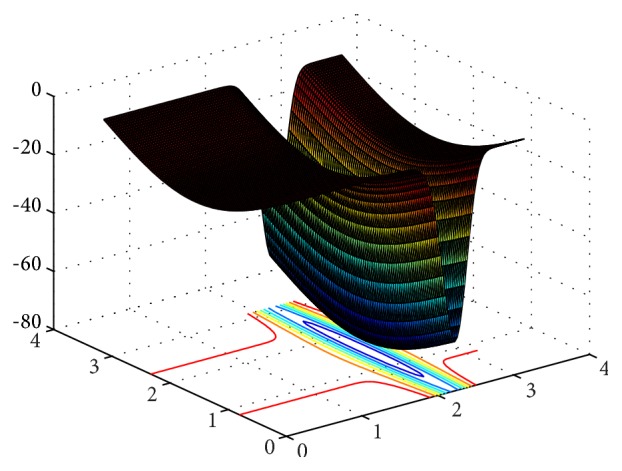
3D surface figure of Michalewicz function.

In order to verify the performances of the improved CS algorithm, six typical continuous test functions are chosen for carrying out the simulation research, meanwhile, which is compare simulation results with ABC, PSO, CS and GCS. These six test functions are shown in Table 1. Their 3D surface figures are shown in Figures 2 [Fig fig3] [Fig fig4] [Fig fig5] [Fig fig6]–7.

Sphere function is a simple unimodal function. Rosenbrock function is an inseparable single mode function, and its global extreme value is in steep valleys. For the most search algorithms, it is difficult to acquire the right search direction within the canyon. Griewank function is a multimodal function with multiple local optimal points, and due to the correlation between variables, it will be very hard to obtain the global optimal solution. Rastrigrin function is a typical inseparable multimodal function, and in its searching domain, there are a large number of local minimum values, which leads to the fact that it is difficult to obtain the global optimum. Michalewicz function has *d*! local extreme values.

The experimental parameters are set as follows. For particle swarm optimization (PSO) algorithm the particle number is *n* = 30; learning factors *c*_1_ = 2, *c*_2_ = 2; the inertia weight *w* = 0.9. For artificial bee colony (ABC) algorithm total number of colonies is *n* = 20, the number of following bees and leading bees is the same *n*/2 = 10. For cuckoo search (CS) algorithm and its 3 improved CS algorithm the total bird's nest population *n* = 25, the detection probability *P*_*a*_ = 0.25, the step length controlled parameter *α* = 0.01. The cuckoo search algorithm based on Gauss disturbance (GCS) and the cuckoo search algorithm based on self-learning and self-evolving disturbance (SSCS) adopt the same disturbance scope control quantity *γ* = 0.75. For the cuckoo search algorithm based on repeat-cycle asymptotic self-learning and self-evolving disturbance (RC-SSCS), the scope of *γ* is [0.25, 1.5], the scale of learning and evolution is set *c*_1_ = *c*_2_ = 0.75. The number of the circulation disturbance *N* = 10. For all algorithms, the dimension of these six test functions is all set *D* = 20. The number of iterations iter = 500. For each algorithm, its program run 30 times independently.

**Figure 8:**
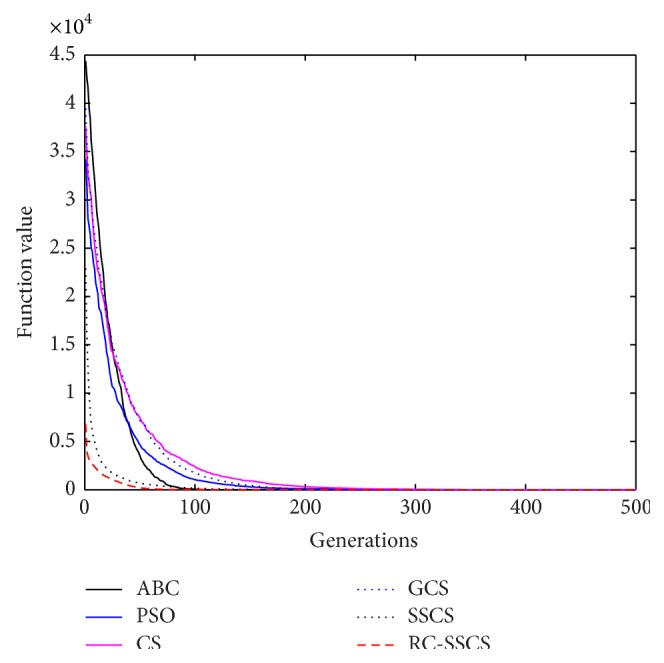
Convergence curves of Function *f*_1_.

**Figure 9 fig9:**
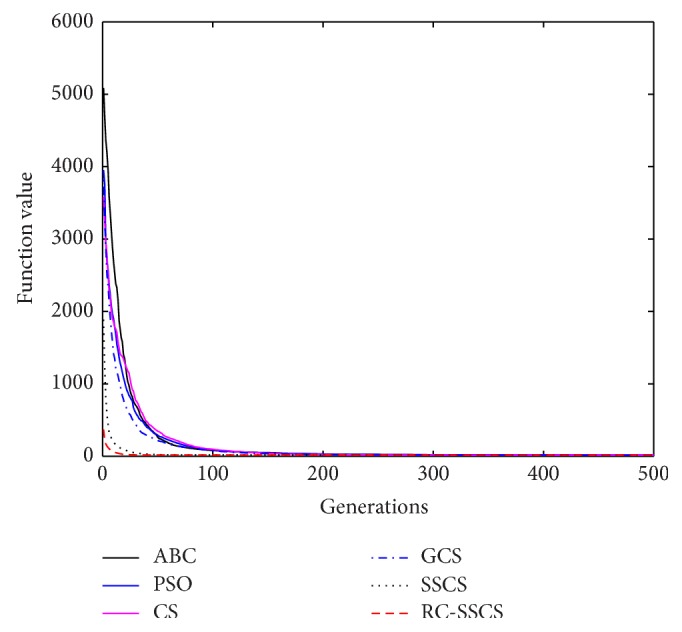
Convergence curves of Function *f*_2_.

**Figure 10 fig10:**
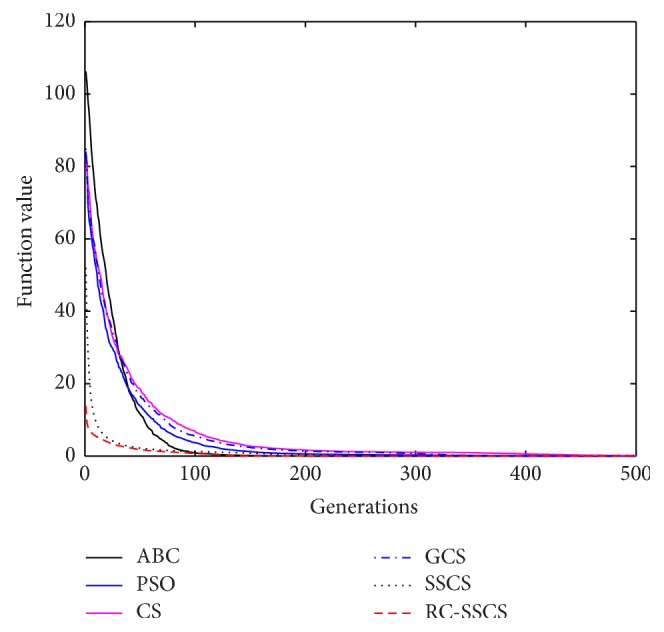
Convergence curves of Function *f*_3_.

**Figure 11 fig11:**
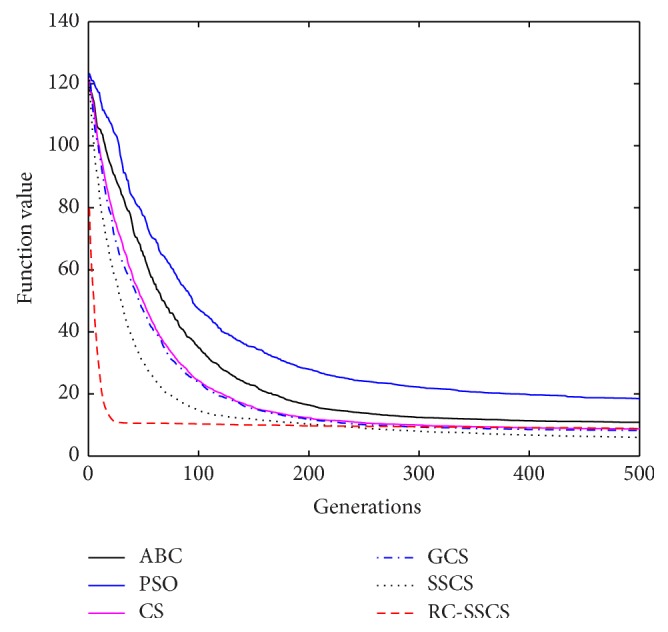
Convergence curves of Function *f*_4_.

**Figure 12 fig12:**
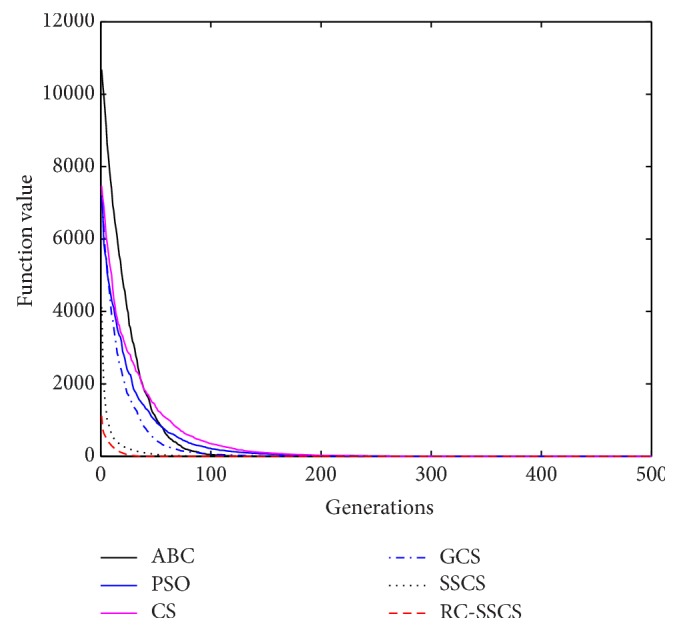
Convergence curves of Function *f*_5_.

**Figure 13:**
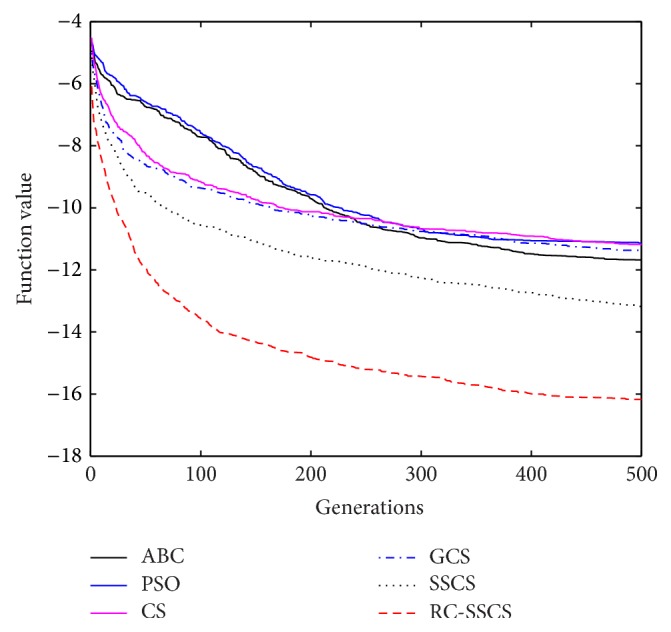
Convergence curves of Function *f*_6_.

Evaluate the performances of algorithms through statistics of the best value, average value and worst value among 30 times running, and the convergence curves of each function. The convergence curves of six functions *f*_1_–*f*_6_ are shown in Figures 8 [Fig fig9] [Fig fig10] [Fig fig10] [Fig fig11] [Fig fig12]–13. The numerical test results of each algorithm are shown in Table 2.

After 500 iterations and 30 times independently running, it can be seen from the convergence curves and numerical statistics results of six functions that the convergence rate and the optimization accuracy of RC-SSCS algorithm is the best. And the convergence rate and optimization accuracy of the two algorithms SSCS and RC-SSCS proposed in this paper are obviously better than the original CS algorithm, GCS algorithm, ABC algorithm, and PSO algorithm.

Seen from six convergence curves, the convergence rate of the six functions is all obviously improved. The convergence curves of function *f*_2_ show that the optimization accuracy achieved by RC-SSCS algorithm after 20 times iteration is equal to that achieved by GCS algorithm and CS algorithm after 200 iterations. The function *f*_3_ convergence curve shows that the optimization accuracy by RC-SSCS algorithm after 100 iterations reached is equal to the accuracy by original CS algorithm after 400 iterations reached. From the convergence curve of function *f*_4_, It can be seen that the optimization accuracy achieved by RC-SSCS algorithm after 25 iterations is equal to that by SSCS algorithm after 175 iterations achieved and by CS algorithm after 300 iterations achieved. The convergence rate of function *f*_5_ and *f*_6_ is also changed obviously. The results show that the improved algorithm makes the convergence rate be greatly improved.

Seen from the numerical results of Table 2, the improved algorithm makes the optimization accuracy of six typical functions be improved. The SSCS algorithm and RC-SSCS algorithm relative to CS algorithm, respectively, make the best value of single-mode function *f*_1_ increased by 5 and 10 orders of magnitude, and the average value, respectively, increased by 4 and 7 orders of magnitude. For multimode function *f*_3_ with multiple local optimal points, compared with the original CS algorithm, SSCS, and RC-SSCS algorithm, respectively, make its best value and average value improved by 5 and 9 orders of magnitude. Optimization accuracy of other functions has been improved. It shows that the improved algorithm can make the optimization accuracy be improved.

**Figure 14:**
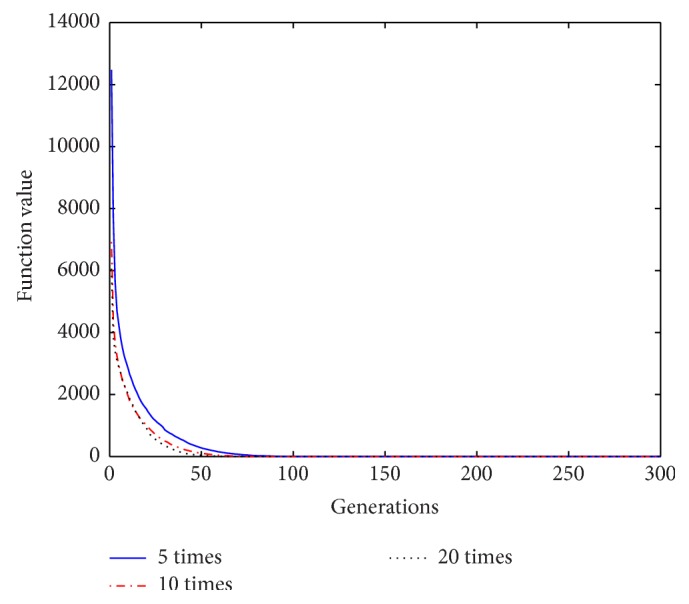
Convergence curves of Function *f*_1_ under different disturbance number.

**Figure 15 fig15:**
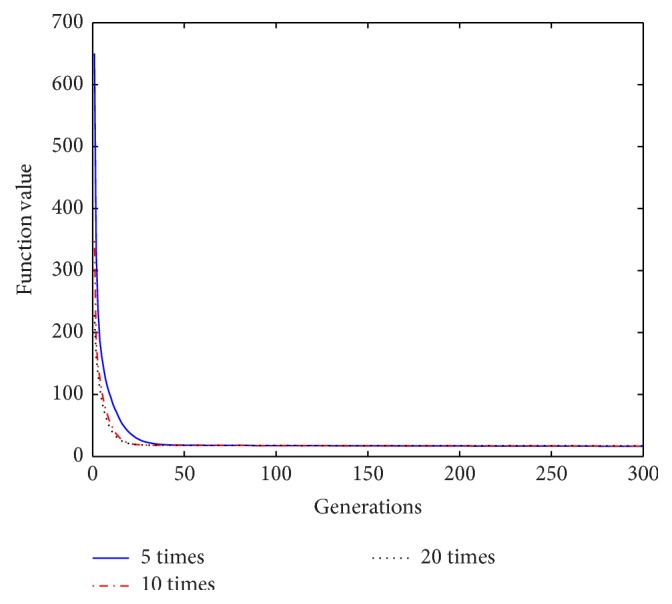
Convergence curves of Function *f*_2_ under different disturbance number.

**Figure 16 fig16:**
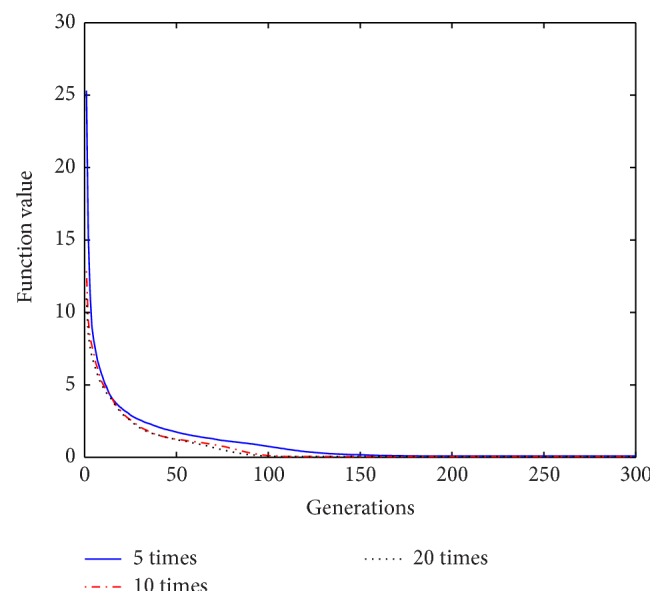
Convergence curves of Function *f*_3_ under different disturbance number.

**Figure 17 fig17:**
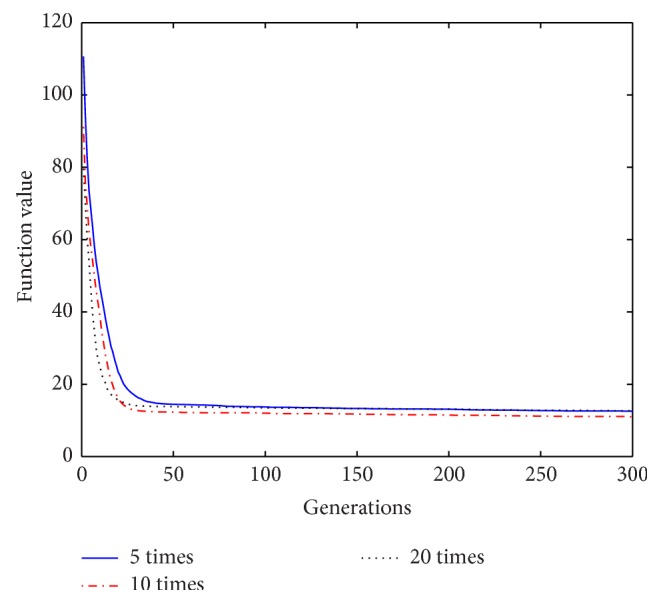
Convergence curves of Function *f*_4_ under different disturbance number.

**Figure 18 fig18:**
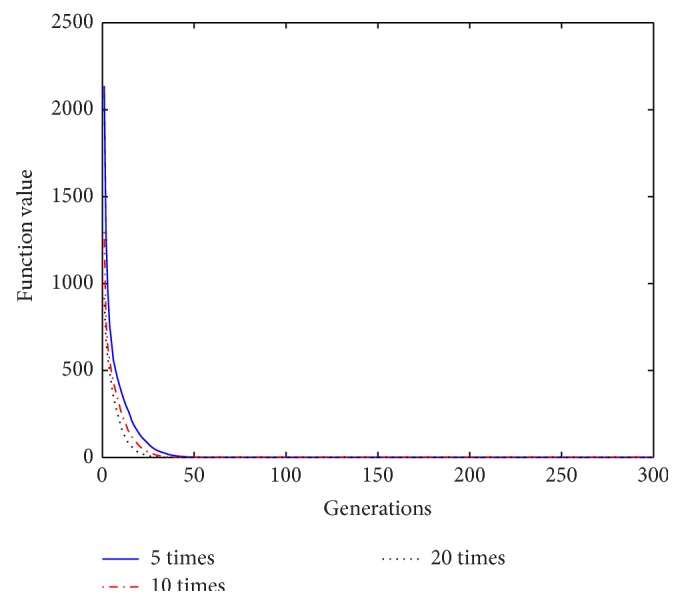
Convergence curves of Function *f*_5_ under different disturbance number.

**Figure 19:**
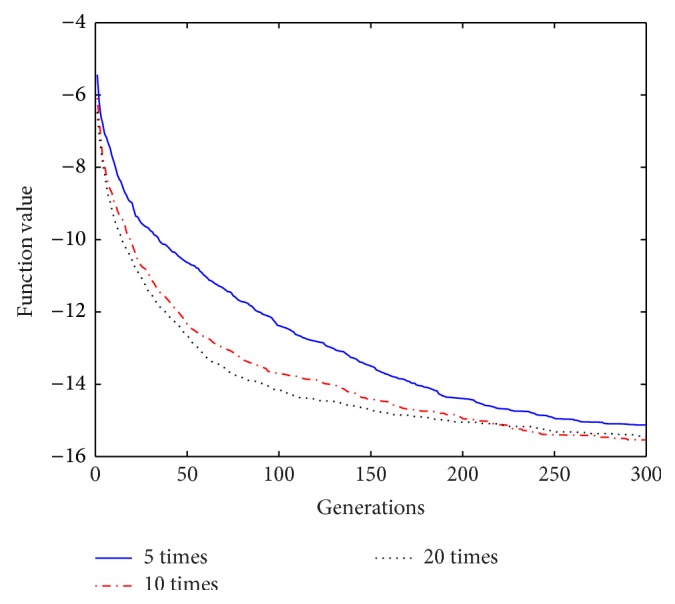
Convergence curves of Function *f*_6_ under different disturbance number.

Based on the above analysis of the improved algorithm, it is known that, within a certain range of disturbance, the number of the repeat-cycle disturbance affects the balance between the search capability and the optimization accuracy in disturbance process. An appropriate disturbance time can make a good balance between search ability and search accuracy and play a best optimization effect. In process of disturbance search, if the algorithm only has strong search ability but no high search precision, it will not get an ideal search effect, Similarly, if the precision is very high, but search ability is weak, it will be also no ideal search effect. In order to select a reasonable disturbance time, in this paper we carry out the further studies on the relationship between the repeat-cycle disturbance times and the convergence rate and optimization precision. The same parameter settings are chosen as described above, except the number of iterations set iter = 300. The cycle disturbances to each function are carried out 5 times, 10 times, and 20 times, respectively. The results of the six functions under different disturbance are shown in Figures 14 [Fig fig15] [Fig fig16] [Fig fig17] [Fig fig18]–19 and Table 3.

It can be seen from the convergence curves in Figures 14–19 that with the increase of cycle's times, the convergence rate of six functions will be gradually improved. But by looking carefully at each function convergence curve, it can be discovered that the changing of convergence rate when number of loops increases from 5 to 10 is bigger than that when loops number increases from 10 to 20. That is to say, the changing of convergence produced by increasing 5 times loops in front of repeat-cycle disturbance is bigger than that by increasing 10 times in later of it. It also can be seen from the numerical results in Table 3 that the optimization accuracy is the highest when cycle disturbance times is 10. The optimization accuracy of six functions is all improved when the disturbance times are increased from 5 to 10. However, when the disturbance times are increased from 10 to 20, the optimization accuracy all decreases instead of increasing.

In conclusion, the more disturbance times may not obtain the better results. Within the same disturbance scope, when the disturbance times reach a certain number, if the disturbance number increases again, the convergence speed of the algorithm does not have an obvious improvement. However, the optimization accuracy will be reduced. Integrally considering the convergence rate, the optimization accuracy and the optimizing time, it is better to choose the cycled disturbance times about 10.

## 5. Conclusion

In order to improve the convergence rate and optimization accuracy of the cuckoo search (CS) algorithm for solving function optimization problems, a kind of cuckoo search algorithm based on the repeat-cycle asymptotic self-learning and self-evolving disturbance (RC-SSCS) is proposed. Six typical test functions are chosen for simulation experiments. Simulation results show the effectiveness of the proposed improved cuckoo search algorithm in convergence rate and optimization accuracy.

## Figures and Tables

**Table 1:** Typical test functions.

Function name	Expression	Scope
Sphere	$f_{1}\left(x\right)=\overset{d}{\underset{i=1}{\sum }}x_{i}^{2}$	[−100,100]
Rosenbrock	$f_{2}(x)=\overset{d-1}{\underset{i=1}{\sum }}\left(100\left(x_{i+1}-x_{i}^{2}\right)+{\left(x_{i}-1\right)}^{2}\right)\;$	[−2.08,2.08]
Griewank	$f_{3}(x)=\frac{1}{4000}\left(\overset{d}{\underset{i=1}{\sum }}\left(x_{i}^{2}\right)\right)-\left(\prod_{i=1}^{d}\cos\left(\frac{x_{i}}{\sqrt{i}}\right)\right)+1$	[−300,300]
Rastrigrin	$f_{4}(x)=\overset{d}{\underset{i=1}{\sum }}\left(x_{i}^{2}-10\cos\left(2\pi x_{i}\right)+10\right)$	[−1.25,1.25]
Sumsquares	$f_{5}(x)=\overset{d}{\underset{i=1}{\sum }}ix_{i}^{2}$	[−10,10]
Michalewicz	$f_{6}(x)=-\overset{d}{\underset{i=1}{\sum }}\sin\left(x_{i}\right){\left[\sin\left(\frac{ix_{i}^{2}}{\pi }\right)\right]}^{2m},(m=10)$	[0, *π*]

**Table 2:** Comparison of numerical testing results.

Function	Algorithm	Best	Worst	Mean
*f*_1_	ABC	3.1519*e* − 004	0.0704	9.0266*e* − 004
	PSO	0.0048	7.0889	0.6284
	CS	0.0382	0.7001	0.2170
	GCS	5.8627*e* − 004	0.0047	0.0016
	SSCS	4.8773*e* − 007	9.1317*e* − 005	1.6997*e* − 005
	RC-SSCS	3.6450*e* − 012	3.0061*e* − 005	1.7847*e* − 008

*f*_2_	ABC	11.3082	18.1271	13.7651
	PSO	10.4223	34.5360	17.4946
	CS	11.1806	17.3151	15.2039
	GCS	8.5219	16.8620	14.2017
	SSCS	6.2653	14.5681	13.3126
	RC-SSCS	3.3432	14.2079	10.8277

*f*_3_	ABC	4.6297*e* − 005	0.6980	0.1859
	PSO	5.8949*e* − 005	1.8793	0.3322
	CS	0.0189	0.3452	0.2010
	GCS	2.7105*e* − 004	0.0077	0.1057
	SSCS	2.2377*e* − 009	0.3909	0.0814
	RC-SSCS	1.0616*e* − 011	0.1954	0.0454

*f*_4_	ABC	5.2919	24.7673	10.3917
	PSO	6.0188	29.7667	18.5742
	CS	5.0454	11.4347	9.4766
	GCS	4.5346	9.4692	8.7840
	SSCS	1.2841	9.3480	6.0351
	RC-SSCS	0.6950	10.3400	5.8859

*f*_5_	ABC	2.5878*e* − 005	8.4891*e* − 004	3.3576*e* − 004
	PSO	5.6717*e* − 004	4.2837	0.2335
	CS	8.7353*e* − 004	0.0068	0.0030
	GCS	1.5714*e* − 004	9.2999*e* − 004	4.2927*e* − 004
	SSCS	9.3420*e* − 007	9.5495*e* − 004	9.8643*e* − 005
	RC-SSCS	2.4677*e* − 009	8.4555*e* − 004	5.6853*e* − 006

*f*_6_	ABC	− 14.7812	− 8.1498	− 11.0840
	PSO	− 15.1576	− 7.8576	− 10.8402
	CS	− 12.7182	− 10.1137	− 11.3479
	GCS	− 13.9297	− 10.4565	− 11.6951
	SSCS	− 15.1779	− 11.4004	− 13.6241
	RC-SSCS	− 18.0889	− 13.1681	− 16.7740

**Table 3:** Comparison of numerical testing results under different cycled disturbance number.

Function	Cycle number	Best	Worst	Mean
*f*_1_	5	1.0950*e* − 006	4.5927*e* − 004	8.4772*e* − 005
	10	8.0914*e* − 008	3.9593*e* − 005	3.3471*e* − 006
	20	8.1817*e* − 007	1.1113*e* − 004	2.4506*e* − 005

*f*_2_	5	15.5346	19.4028	18.2092
	10	14.5140	19.4748	16.6741
	20	14.9356	19.4993	16.6707

*f*_3_	5	2.0991*e* − 007	1.2703	0.1498
	10	1.0955*e* − 008	0.3909	0.0847
	20	3.1836*e* − 008	1.3680	0.0847

*f*_4_	5	5.9698	16.9143	12.4617
	10	1.9899	14.9247	11.2695
	20	1.9900	17.9210	11.7947

*f*_5_	5	1.5066*e* − 007	1.3715*e* − 004	5.1063*e* − 005
	10	5.5967*e* − 008	0.0052	3.6228*e* − 005
	20	3.2597*e* − 007	0.0022	2.9710*e* − 004

*f*_6_	5	− 17.3839	− 11.9059	− 15.1020
	10	− 18.2436	− 12.5260	− 15.5739
	20	− 18.0307	− 13.0349	− 15.5256
